# 

**Published:** 1873-04

**Authors:** 


### CONTENTS.

### COMMUNICATIONS.

| | PAGE. |
|---|---|
| Gifts and Gifts. Ben. H. Pratt, A. M. | 112 |
| Renewal of Life. P. H. Hayes, M. D. | 113 |
| Doctors. Dr. Ned | 114 |
| The woman Question. D. L. D. Sheldon, M. D. | 116 |

### CLIPPINGS.

| | |
|---|---|
| Medical Preaching | 116 |
| How to Sleep | 134 |
| Experiments on Frogs | 134 |

### CORRESPONDENCE.

| | |
|---|---|
| Twelve Letters, with remarks | 125 |

### EDITORIAL.

| | PAGE |
|---|---|
| Medical Items and News | 117 |
| Household Hints and Recipes | 127 |
| Late | 129 |
| IX Year | 129 |
| Pterygium | 130 |
| The Code | 131 |
| Heating Apparatus | 131 |
| House Drains | 134 |
| Chit-Chat | 135 |
| Books and Periodicals | 138 |

